# Stable expression of *Mycobacterium bovis* antigen 85B in auxotrophic *M. bovis* bacillus Calmette-Guérin

**DOI:** 10.1590/0074-02760160360

**Published:** 2017-02

**Authors:** Caroline Rizzi, Ana Carolina Peiter, Thaís Larré Oliveira, Amilton Clair Pinto Seixas, Karen Silva Leal, Daiane Drawanz Hartwig, Fabiana Kommling Seixas, Sibele Borsuk, Odir Antônio Dellagostin

**Affiliations:** 1 Universidade Federal de Pelotas, Centro de Desenvolvimento Tecnológico, Núcleo de Biotecnologia, Programa de Pós-Graduação em Biotecnologia, Pelotas, RS, Brasil; 2 Universidade Federal de Pelotas, Instituto de Biologia, Departamento de Microbiologia e Parasitologia, RS, Brasil

**Keywords:** bovine tuberculosis, recombinant BCG, auxotrophic complementation, foreign antigens

## Abstract

**BACKGROUND:**

Bovine tuberculosis (TB) is a zoonotic disease caused by *Mycobacterium bovis*, responsible for causing major losses in livestock. A cost effective alternative to control the disease could be herd vaccination. The bacillus Calmette-Guérin (BCG) vaccine has a limited efficacy against bovine TB, but can improved by over-expression of protective antigens. The *M. bovis* antigen 85B demonstrates ability to induce protective immune response against bovine TB in animal models. However, current systems for the construction of recombinant BCG expressing multiple copies of the gene result in strains of low genetic stability that rapidly lose the plasmid in vivo. Employing antibiotic resistance as selective markers, these systems also compromise vaccine safety. We previously reported the construction of a stable BCG expression system using auxotrophic complementation as a selectable marker.

**OBJECTIVES:**

The fundamental aim of this study was to construct strains of *M. bovis* BCG Pasteur and the auxotrophic *M. bovis* BCG Δ*leuD* expressing Ag85B and determine their stability in vivo.

**METHODS:**

Employing the auxotrophic system, we constructed rBCG strains that expressed *M. bovis* Ag85B and compared their stability with a conventional BCG strain in mice. Stability was measured in terms of bacterial growth on the selective medium and retention of antigen expression.

**FINDINGS:**

The auxotrophic complementation system was highly stable after 18 weeks, even during in vivo growth, as the selective pressure and expression of antigen were maintained comparing to the conventional vector.

**MAIN CONCLUSION:**

The Ag85B continuous expression within the host may generate a stronger and long-lasting immune response compared to conventional systems.

Bovine tuberculosis (TB), which is caused by *Mycobacterium bovis*, represents a major economic and animal health problem for the farming community. The zoonotic potential of bovine TB remains a concern in countries with few or no control policies (Phillips et al. 2003, Thoen et al. 2009). Cattle vaccination is an inexpensive method of control and perhaps the only one able to eradicate the disease, especially where bovine TB is endemic (Waters et al. 2012, Conlan et al. 2015, Vordermeier et al. 2016). The only currently available vaccine candidate, *M. bovis* bacillus Calmette-Guérin (BCG), does not induce a high level of protection against bovine TB (Hewinson et al. 2003). A comparison of *M. bovis* and BCG genomes has shown that different regions of the BCG chromosome have been deleted relative to those of the parental strain (Behr et al. 1999). These deletions produced have resulted in an attenuated strain and may have eliminated potentially protective immune antigens (Lewis et al. 2003), which could explain the lack of efficacy of this vaccine in cattle (Khare et al. 2007).

On the other hand, the BCG vaccine has been used to immunise more than two billion individuals against TB, with a long record of effectiveness and safety in humans. Its adjuvant properties can elicit both humoral and cell-mediated immune responses. Additionally, BCG is inexpensive to produce, can be given at any time after birth, and is not affected by maternal antibodies. In addition, it is one of the most heat-stable live vaccines (Stover et al. 1991, Singh et al. 2016). BCG has commonly been employed as a delivery system to express heterologous mycobacterial genes (da Costa et al. 2014), as well as genes from other bacteria, viruses, and mammalian cells (Singh et al. 2016).

One candidate vaccine against bovine TB is BCG expressing the *M. bovis* immunodominant protein known as antigen 85B (Ag85B). *M. tuberculosis* Ag85B is abundantly expressed by bacteria in infected human monocytes and is involved in the synthesis of the mycobacterial cell wall (Wiker & Harboe 1992, Harth et al. 1996). In *M. bovis*, Ag85B is encoded by *fbpB*, which shows high identity with the *M. tuberculosis* antigen (Harth et al. 1996). An rBCG secreting *M. tuberculosis* Ag85B was the first vaccine shown to be more potent against *M. tuberculosis* than conventional BCG (Horwitz & Harth 2003). Another rBCG expressing *M. bovis* Ag85B induced greater protective immunity than BCG against aerosol challenge with a highly virulent strain of *M. bovis* in guinea pigs (Horwitz et al. 2006).

Genetically stable strains are of particular importance for rBCG vaccines because continuous and enhanced expression has been shown to elicit a stronger immune response (Himmelrich et al. 2000, Méderlé et al. 2002). We previously developed an expression system employing a leucine auxotrophic BCG mutant (BCG Pasteur Δ*leuD*) and a replicative vector (pUP410) that complements this mutation (Borsuk et al. 2007). Auxotrophic BCG strains are unable to access amino acids when inside macrophages; thus, they fail to grow in vivo (Bange et al. 1996). This auxotrophic complementation system allowed the production of stable recombinant strains in vivo, with high levels of recombinant protein expression and without the use of an antibiotic resistance marker (Borsuk et al. 2007, Seixas et al. 2010).

Employing the auxotrophic system, we constructed rBCG strains that expressed *M. bovis* Ag85B. An evaluation of the immune response induced by an rBCG/Ag85B strain was performed in cattle (Rizzi et al. 2012), and the use of rBCG/Ag85B as an immunotherapy for bladder cancer was examined in an in vitro study (Begnini et al. 2013); in both studies, the protection levels by rBCG/Ag85B were higher than those elicited by the conventional BCG Pasteur vaccine, but stability studies were not performed. In this study, we compared the stability of the constructs using a conventional BCG strain and the auxotrophic strain. The auxotrophic strain tested retained the vector functional integrity during in vivo passages, as demonstrated by expression levels in bacteria recovered from mice.

## MATERIALS AND METHODS

*Ethics statement* - Animal experiments were performed inside the biosafety facilities of the Federal University of Pelotas (UFPel), Brazil, in compliance with the regulations of the Ethics Committee on Animal Experimentation (CEEA) of UFPel. Ethics approval for the study was obtained from CEEA (n. 9412).

*Bacterial strains and culture conditions* - *Escherichia coli* strains TOP10 (Invitrogen) and BL21 Star (DE3) (Invitrogen) were grown in Luria-Bertani medium at 37ºC with the addition of kanamycin 50 µg mL^-1^ or ampicillin 100 µg mL^-1^, respectively. *M. bovis* BCG Pasteur and BCG Pasteur ∆*leuD* were grown in Middlebrook 7H9 broth (Difco) supplemented with 10% oleic acid, albumin, dextrose complex (OADC, Difco), 0.2% glycerol, and 0.05% Tween 80 (Sigma), or in 7H10 agar (Difco) containing 10% OADC and 0.2% glycerol. When necessary, BCG strains were grown in media supplemented with 100 µg mL^-1^ L-leucine (Sigma) or 25 µg mL^-1^ kanamycin (Sigma).

*Production of homogeneous Ag85B* - Synthetic oligonucleotides used for polymerase chain reaction (PCR) amplification of *fbp*B were designed based on the complete genome sequence of *M. bovis* AF2122/97 and using Vector NTI 10.0 software (Invitrogen) (Table). Both forward and reverse primers contained restriction sites, which are underlined (Table). A portion of *fbpB* (875 bp) was amplified from genomic DNA using standard PCR conditions and GoTaq Hot Start Polymerase (Promega) (Rizzi et al. 2012). The PCR product was digested with *Bam*HI and *Hind*III enzymes (Promega) and inserted into the pAE vector (Ramos et al. 2004), which had been previously digested with the same restriction enzymes. Competent *E. coli* TOP10 cells were transformed with the ligation product, and clones were verified by restriction enzyme digestion and PCR. The recombinant vector (pAE::85B) was transformed into *E. coli* BL21 Star (DE3), (Invitrogen) and the protein expression was induced with 1 mM isopropyl β-D-1-thiogalactopyranoside (IPTG). Cells were lysed by sonication and expression in the cell fractions was analysed by 15% sodium dodecyl sulphate-polyacrylamide gel electrophoresis (SDS-PAGE) with Coomassie blue staining. Purification of recombinant Ag85B (rAg85B) was performed by affinity chromatography on a Ni-sepharose column using the automated liquid chromatography system ÄKTAprime (GE Healthcare). The protein was purified using buffer containing a chaotropic agent (100 mM Tris-HCl, 300 mM NaCl, 6 M urea, pH 8.0), dialysed in 100 mM Tris-HCl, pH 8.0 for refolding, and concentrated by ultrafiltration using an Amicon ultrafiltration cell (MW 30,000 Da). The rAg85B protein was characterised by western blot employing a His-tag monoclonal antibody (Sigma-Aldrich). Homogeneous protein was quantified by the BCA method (BCA Protein Assay Kit, Thermo Scientific) and stored at -80ºC.

**TABLE:** Strains, primers, and restriction enzymes used for cloning of various portions of *Mycobacterium bovis fbpB* and its protein products

Strains used for expression	Primer pairs^a^	Restriction enzymes^b^	Recombinant vectors obtained
*Escherichia coli* BL21 Star (DE3)	Forward primer: 5’tataagctttcagccggcgcc3’ Reverse primer: 5’atggatccttctcccggccg3’	*Bam*HI *Hind*III	pAE::85B
BCG Pasteur and BCG *∆leuD*	Forward primer: 5’ggggtacccgctatgtagctccaattc3’ Reverse primer: 5’ggggtacctcagccggcgcc3’	*Kpn*I	pUP410::85B
*E. coli* Top 10	Forward primer: 5’gttctagacttctcccggccg3’ Reverse primer: 5’tataagctttcagccggcgcc3’	*Xba*I *Hin*dIII	pUS2000::85BT
BCG Pasteur and BCG ∆*leuD*	Forward primer: 5’ggggtacccgctatcgtagactc3’ Reverse primer: 5’ggggtacctcagccggcgcc3’	*Kpn*I	pUP410::85BT

*a*: the restriction enzymes cleavage sites are underlined; *b*: enzymes employed to digest the amplified fragments and their respective vectors during the cloning process; BCG: bacillus Calmette-Guérin.

*Production of polyclonal antibodies* - Two six-week-old female BALB/c mice were inoculated intraperitoneally with 100 µg of r85B in incomplete Freund’s adjuvant. Booster doses were injected two and three weeks after the first inoculation. Thirty days after inoculation, the animals were euthanised using sodium pentobarbital, and peripheral blood was collected by cardiac puncture to obtain hyperimmune serum. Antibody responses were monitored by indirect enzyme-linked immunosorbent assay (ELISA) using puriﬁed rAg85B. Polystyrene plates were coated overnight with 250 ng of rAg85B per well, followed by incubation with serial dilutions of sera for 1 h at 37ºC. Peroxidase-conjugated anti-mouse immunoglobulin G was added, and the resulting reaction was visualised using *o*-phenylenediamine dihydrochloride (Sigma) and hydrogen peroxide. Absorbances were determined at 450 nm, and mean values were calculated from serum samples assayed in duplicate. The rAg85B protein was characterised by western blot employing polyclonal antibody anti r85B.

**Fig. 1:**
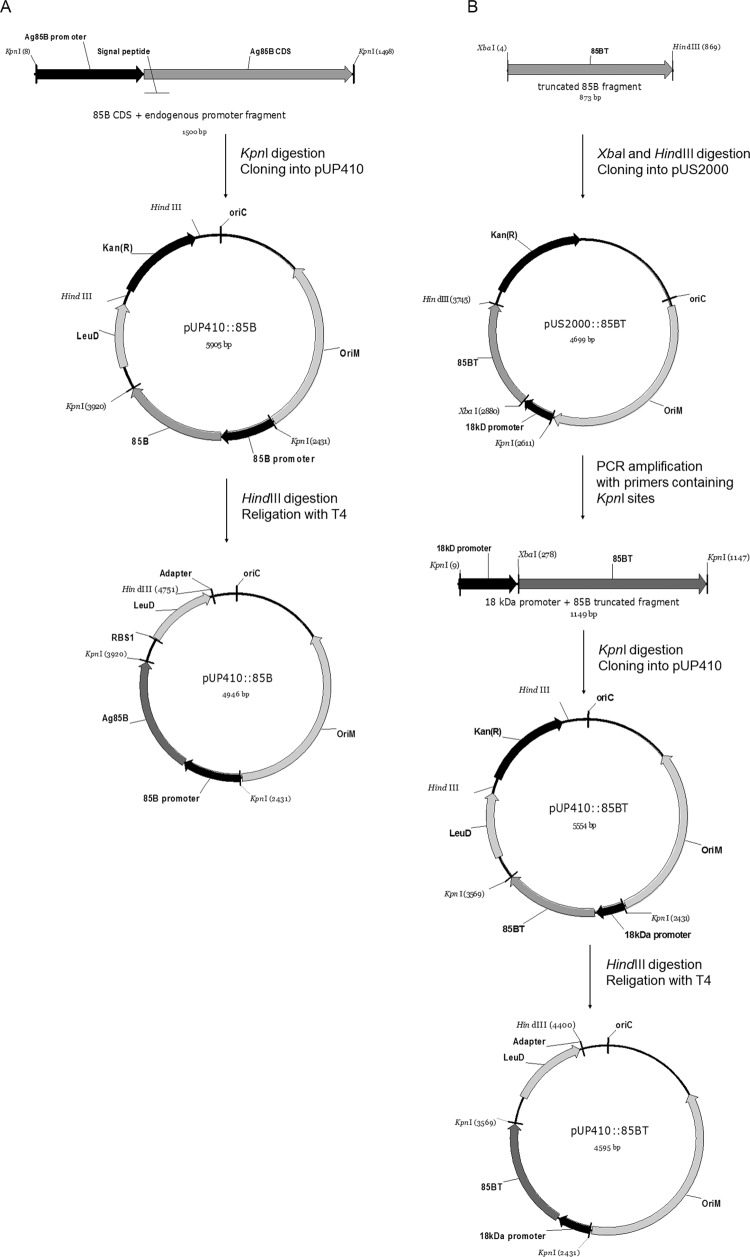
: schematic outline of the process of constructing recombinant plasmids for Ag85B expression in *Mycobacterium bovis* bacillus Calmette-Guérin. The DNA fragments cloned into vector pUP410 were previously amplified by polymerase chain reaction (PCR) from *M. bovis* genomic DNA. (A) Cloning of the *fbp*B coding region and its endogenous promoter, resulting in pUP410::85B; (B) cloning of a portion of *fbpB* that encodes the mature protein. First, a portion of the *fbpB* coding region (120 to 978 bp) was cloned into vector pUS2000, yielding pUS2000::85BT. From the resulting recombinant vector, an 1149-bp amplicon containing the cloned fragment under control of an 18-kDa promoter from *M. leprae* was obtained by PCR and then cloned into pUP410, yielding pUP410::85BT. The kanamycin resistance gene was removed by digestion with the *Hind*III enzyme, and the digestion product was ligated using the T4 ligase enzyme.

*Cloning of M. bovis fbpB in the mycobacterial expression vector* - Synthetic oligonucleotide primers were used for PCR amplification of the total and partial *fbp*B (Table). These pair of primers were used to amplify the *M. bovis* 85B gene cassette, consisting of *fbpB* (1500 bp) and a fraction of the coding region (873 bp). The two different fragments of *fbpB* were cloned under the control of different promoters into the pUP410 vector, as shown in Fig. 1. The first construct (pUP410::85B) comprised the *fbpB* coding region, which encodes the secretion signal peptide and endogenous promoter. The second construct (pUP410::85BT) constituted the mature protein coding sequence, i.e., the sequence without that encoding the signal peptide, placed under the control of the *M. leprae* 18-kDa promoter (Dellagostin et al. 1995). The fragments were amplified from genomic DNA as described above. Then, the PCR products were digested with restriction enzymes, and the fragments were inserted into pUS2000, which was used as a template for further amplification of the 18-kDa promoter and the pUP410 mycobacterial expression vector (Fig. 1) (Borsuk et al. 2007). Competent *E. coli* TOP10 cells were then transformed with the recombinant plasmids, and the clones were verified by restriction enzyme digestion and PCR. In plasmids employed for the transformation of BCG Δ*leuD* strains, the kanamycin resistance gene was removed after cloning. The recombinant plasmids were digested with the *Hind*III enzyme, directed as sites flanking both ends of the kanamycin resistance gene, and re-ligated using the T4 ligase enzyme (Fig. 1).

*Construction of recombinant BCG* - Electrocompetent BCG strains (BCG Pasteur and BGC Pasteur ∆*leu*D) were transformed with pUP410::85B and pUP410::85BT as described by Parish and Stoker (1995). The recombinant strains were selected in 7H10 media containing 25 µg mL^-1^ of kanamycin or without L-leucine supplementation. BCG transformants were grown for five days in selective 7H9 liquid media, and 10 mL of culture volume was adjusted to a concentration of 10^9^ cells and centrifuged (4000 × *g* for 10 min). The resulting pellet was suspended in 1 mL of 100 mM Tris, pH 8.0, and the cells were lysed using a Ribolyser (Hybaid). The lysate was then centrifuged (14000 × *g* for 10 min), and the supernatant was recovered. Saturated ammonium sulphate solution was added to the supernatant in order to precipitate the secreted recombinant protein, which was then collected by centrifugation (4000 × *g* for 10 min). Expression of the recombinant proteins was demonstrated by western blot. Proteins in the cell lysate and culture supernatant were separated by 15% SDS-PAGE and electrotransferred to a nitrocellulose membrane (GE Healthcare Live Sciences). Blots were probed with mouse polyclonal anti-85B antibody. Peroxidase-conjugated anti-mouse immunoglobulin G (Sigma-Aldrich) was used at a dilution of 1:6000. Proteins were detected using the TMB Liquid Substrate System (Sigma-Aldrich). Recombinant BCG liquid cultures were also used to prepare the inoculum. Bacterial cultures were grown to an optical density at 600 nm (OD_600_) of 0.6, centrifuged at 4000 × *g*, and suspended in an appropriate volume of sterile phosphate buffered saline (PBS).

*Inoculation and in vivo recombinant vector stability* - Groups of seven six-week-old female BALB/c mice were inoculated intraperitoneally with 10^6^ colony forming units (CFU) of BCG Pasteur-85B, BCG Pasteur-85BT, ∆*leu*D BCG-85B, or ∆*leu*D BCG-85BT. The animals were euthanised with sodium pentobarbital at either eight or 18 weeks after inoculation. Spleens were removed from animals under aseptic conditions, macerated in PBS (pH 7.2), and filtered. After centrifugation (2000 × *g* for 5 min), the tissue was suspended in PBS with 0.1% Tween 80 and centrifuged again (14000 × *g* for 10 min). The tissue was then suspended in Middlebrook 7H9 medium (Difco) and serially diluted. The dilutions were plated on 7H10 solid medium supplemented with 10% OADC (Difco) in the presence or absence of selective markers (kanamycin for BCG Pasteur-85B and BCG Pasteur-85BT, and L-leucine for ∆*leuD* BCG-85B and ∆*leuD* BCG-85BT) and incubated at 37ºC for 30 days. The in vivo stability of the constructs was determined by counting the number of colonies on each plate and calculating the percentage of bacteria that retained the recombinant vector, indicated by bacterial growth on the selective medium. The data was statistically analysed using a paired *t*-test, and p < 0.05 was considered significant. The recovered colonies were grown in supplemented 7H9 medium in the presence of selective markers. Ag85B expression was analysed by western blot as described above.

## RESULTS

**Fig. 2:**
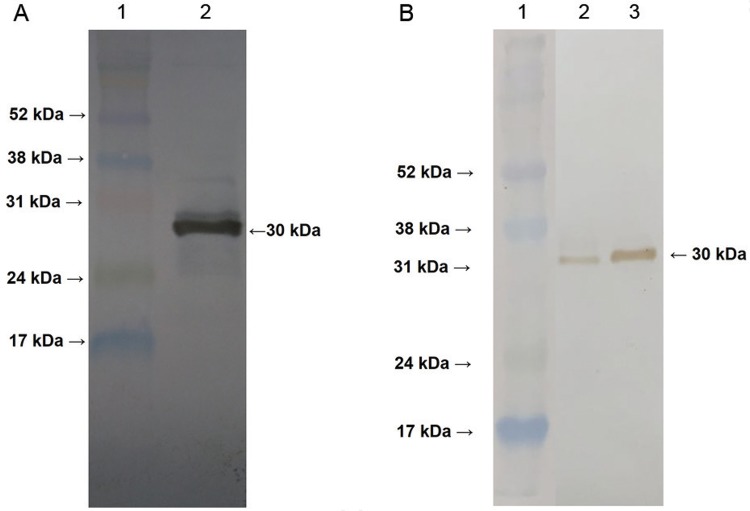
: western blot using a 6× His-tag monoclonal antibody or polyclonal anti-r85B antibody to confirm the identity of rAg85B. (A) rAg85B was overexpressed in *Escherichia coli* Star (DE3), purified using a nickel affinity column, and identified by 6× His-tag monoclonal antibody. Lane 1, Full-Range Rainbow Molecular Weight Protein Marker (GE); lane 2, purified rAg85B. (B) rAg85B was recognised by polyclonal anti-r85B antibody produced in mice. Lane 1, Full-Range Rainbow Molecular Weight Protein Marker (GE); lane 2, purified rAg85B after refolding; lane 3, purified rAg85B.

*Production of homogeneous Ag85B and polyclonal antibodies* - To detect the recombinant antigen 85B (rAg85B) in mycobacterial cultures, it was necessary to produce polyclonal antibodies in mice. To obtain hyperimmune serum, we initially produced rAg85B in *E. coli* and then inoculated the protein into BALB/c mice. The gene fragment encoding the mature Ag85B protein was successfully amplified by PCR and cloned into the pAE vector, resulting in vector pAE::85B. After IPTG induction, *E. coli* BL21 Star (DE3) cells transformed with pAE::85B showed expression of a recombinant protein with the expected molecular mass (~30 kDa), although it was found in the insoluble fraction. Thus, rAg85B was purified using a nickel affinity column with 6 M urea and then dialysed for refolding. One litre of cell culture suspension yielded approximately 6 mg of homogeneous rAg85B. The identity of the purified protein was confirmed by western blot using a 6× His-tag monoclonal antibody (Fig. 2A) and the polyclonal antibody anti r85B (Fig. 2B).

**Fig. 3:**
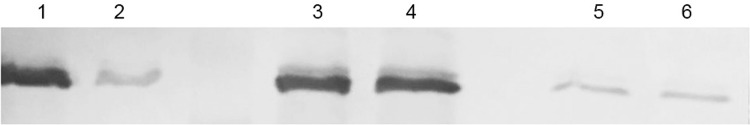
: western blot employing polyclonal anti-r85B antibody to confirm r85B expression in *Mycobacterium bovis* bacillus Calmette-Guérin (BCG) strains. BCG ∆*leuD* expression profile. Lanes 1 and 2, whole-cell lysates and culture supernatant, respectively, of rBCG transformed with pUP410::85BT; lanes 3 and 4, whole-cell lysates and culture supernatant of rBCG transformed with pUP410::85B; lanes 5 and 6, whole-cell lysates and culture supernatant of wild-type BCG.

*Production of BCG strains expressing Ag85B* - Recombinant BCG strains transformed with pUP410::85B and pUP410::85BT were evaluated by western blot. We observed the presence of recombinant Ag85B with the expected molecular mass (~30 kDa) in the supernatant of whole-cell lysates (Fig. 3). However, only ∆*leuD* BCG + pUP410::85B (∆*leuD* BCG-85B) showed Ag85B secretion into culture supernatant (Fig. 3).

**Fig. 4:**
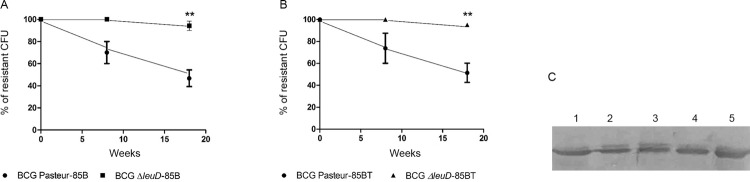
: in vivo stability of rBCG transformed with vectors containing auxotrophic complementation. Mice were inoculated with bacillus Calmette-Guérin (BCG) ∆*leuD-*85B, BCG ∆*leuD*-85BT, BCG Pasteur-85B, or BCG Pasteur-85BT. Bacteria were recovered from spleens, eight and 18 weeks after inoculation and plated on selective and non-selective media. (A-B) Ratios of resistant versus total BCG colonies by spleen were calculated for each strain and construction tested. Vertical bars indicate standard deviation values, and significance was determined by paired *t*-test: ** Statistical significance, p < 0.01. (C) Western blot demonstrating r85B expression in BCG strains recovered from spleens: line 1, BCG Pasteur-85B; line 2, BCG Δ*leuD-*85B; line 3: BCG Δ*leuD*-85BT; lines 4 and 5, positive control strains BCG Δ*leuD-*85B and BCG Δ*leuD-*85BT, respectively, before inoculation.

*In vivo analysis of rBCG stability* - The stability of *M. bovis* BCG ∆*leuD* and *M. bovis* BCG Pasteur was evaluated in mice. The BCG ∆*leuD* strains were transformed with recombinant pUP410 without the kanamycin resistance gene because this plasmid has the complementary gene *leuD*, and this complementation allows the selection of transformants during rBCG construction (Construction of recombinant BCG). However, removal of the kanamycin resistance gene from the recombinant pUP410 vectors used to transform the BCG Pasteur strain was not possible, because resistance to kanamycin is needed to select the transformed strains. Then, the retention of recombinant pUP (strain stability) was determined by counting the numbers of cells growing in the respective selective medium (without leucine for BCG ∆*leuD* strain and with kanamycin for the BCG Pasteur strain). The recombinant pUP410 vector showed stabilities of 94.24% (SD: 1.34) and 95.5% (SD: 1.64) for BCG Δ*leuD-*85B and BCG Δ*leuD*-85BT, respectively, over the 18 weeks of the experiment. In contrast, about 46.8% (SD: 16.99) of BCG Pasteur-85B and 51.4% of BCG Pasteur-85BT (SD: 19.7) cells lost the recombinant vector during the same period (p < 0.001) (Fig. 4A-B). The western blot analysis showed the expression of recombinant protein in bacteria recovered from spleens, confirming the functional stability of the recombinant strains (Fig. 4C).

## DISCUSSION

The unique characteristics of BCG, such as its immunomodulatory properties and ability for a single dose to trigger long-lasting immunity have allowed its successful use as a vaccine vector expressing heterologous antigens. In vivo genetic stability is of special importance in the use of live bacterial vaccines. Antigen expression is an important factor for an effective recombinant bacterial vaccine; expression in vivo has to last for a period long enough to induce a protective response in the host. rBCG can be obtained using two distinct genetic systems for heterologous gene expression: integrative vectors and episomal vectors. Integrative vectors, derived from temperate mycobacteriophages, integrate genes into specific sites in the bacterial chromosome, and when no excision functions operate, the vector can be stably maintained (Machowski et al. 2005). Although this strategy allows the persistent expression of foreign antigens in vivo, these rBCGs showed low levels of expression because only a single copy of the heterologous gene was present in the mycobacterial genome (Méderlé et al. 2002). Episomal vectors are present in five or more copies per transformed cell (Ranes et al. 1990), show high levels of expression, and elicit strong immune responses against heterologous antigens (Dennehy & Williamson 2005). However, the absence of selective pressure for the episomal vector after the vaccine is injected allows the vector to be lost (Dennehy & Williamson 2005). This may compromise the stability of the rBCG and, consequently, the establishment of long-term immune memory (Méderlé et al. 2002).

In this study, we developed rBCGs overexpressing *M. bovis* Ag85B using an auxotrophic complementation system that allowed stable in vivo expression of the recombinant proteins. This system, consisting of an auxotrophic strain unable to synthesise leucine and demonstrating vector complementation, abolishes the need to use an antibiotic resistance gene as a selective marker (Borsuk et al. 2007). Moreover, because leucine auxotrophic BCG is unable to multiply within macrophages (Bange et al. 1996), complementation by the vector allows active selection in vivo.

We investigated whether rBCG ∆*leuD* transformed with recombinant vectors containing and demonstrating auxotrophic complementation is more stable in vivo than rBCG Pasteur. Our results showed that, in mice, rBCG ∆*leuD* is highly stable (93%) for at least 18 weeks, because due to selective pressure is maintained by the auxotrophic system (Borsuk et al. 2007). In contrast, during the same period, about half of the recombinant Pasteur strain transformed with the same vector had lost the recombinant vector. Furthermore, the in vivo stability of the ∆*leuD* rBCG constructs has already been shown for β-galactosidase (Borsuk et al. 2007) and for LipL32 and LigAni antigens of *Leptospira interrogans* (Seixas et al. 2010). Hart et al. (2015) evaluated the retention of plasmid, antigen expression, and immunogenicity of a BCG leucine auxotrophic system expressing lentiviral antigens. Antigens were persistently expressed in vivo for at least 60 days, which might have contributed to the induction of a long-lasting immune response (Hart et al. 2015).

It is important to emphasise that kanamycin resistance is not necessarily a reliable indicator of functional stability and antigen production by the recombinant strain. Several studies that have evaluated in vivo stability of recombinant BCG have shown that bacteria that retain their antibiotic resistance ability had lost the ability of express the recombinant antigen, as reviewed by Dennehy and Williamson (2005). In our study, all auxotrophic strains tested maintained the vector’s functional integrity during in vivo passage, as demonstrated by expression levels in bacteria recovered from the spleen (Fig. 4C). Méderlé et al. (2002) showed that a genetically stable recombinant BCG induced higher levels of protection because of the persistence of bacteria within antigen presenting cells (APCs) that constantly released the recombinant protein. BCG ∆*leuD*-85B has been evaluated as a vaccine against bovine TB and has been shown to generate a strong and long-lasting immune response, (Rizzi et al. 2012), possibly due to the continuous expression of antigen 85B in hosts. Persistent expression of the antigen has been shown to be more immunogenic in animal models. Yu et al. (2011) observed that higher levels of expression induced a stronger immunogenicity in mice than when recombinant mycobacteria with lower levels of expression was used.

Variation in stability and expression is also attributed to promoter properties. Strong promoters such as the BCG *hsp60* promoter have been found to express heterologous antigens constitutively, imposing a metabolic burden (Al-Zarouni & Dale 2002). A portion of the host bacterium’s energy and materials is required to maintain the vector and to express the foreign gene. The extent of this burden determines the degree to which fitness of the rBCG is compromised, resulting in the loss of the inserted element or its expression in the bacterial population (Dennehy & Williamson 2005). Aware of this problem, we placed Ag85B expression in BCG under the control of the endogenous or *M. leprae* 18-kDa antigen promoter. The Ag85B endogenous promoter did not appear to induce metabolic burden when used with high copy number mycobacterial expression vectors (Harth et al. 2004). The *M. leprae* 18-kDa promoter weakly expresses heterologous antigens in vitro, but strongly induces these antigens in macrophages, resulting in high expression levels (Dellagostin et al. 1995).

Comparative studies have shown that rBCGs secreting heterologous antigens are able to induce stronger immune responses and better protection than the same antigens expressed in cytoplasmic form. Antigen secretion or fusion to mycobacterial surface lipoproteins allow these antigens access to the class I major histocompatibility complex (MHC) pathway and subsequently enhance immunogenicity. Antigen secretion also prevents accumulation of heterologous proteins in the bacterial cytoplasm, which can become toxic to rBCG (Dennehy & Williamson 2005). The stability of strain BCG ∆*leuD*-85B may have also been supported by the secretion of the antigen, because the heterologous gene encoded a signal peptide for protein secretion.

In this study, we developed two stable strains (BCG *ΔleuD*-85B and BCG *ΔleuD*-85BT) using a mycobacterium auxotrophic system. The stability of these strains was demonstrated by the persistence of the plasmid and recombinant protein expression for a long period after inoculation in mice. Our system maintained selective pressure in vivo, allowing high levels of heterologous antigen expression*.* Further experiments will allow a determination of differences in the immune response induced by the auxotrophic and conventional systems, as well as differences between cytoplasmic and secreted recombinant antigens. We conclude that the auxotrophic system was responsible for the stability of the recombinant strains. Moreover, our system allows the removal of the kanamycin resistance gene as a selective marker, which increases vaccine safety.
